# Supplementary material for: Development and validation of the Self-Awareness of Ego-Threatening Biases Questionnaire (SAETBQ)
Source: PLoS One. 2025 Jul 16;20(7):e0327989. doi: 10.1371/journal.pone.0327989 (PMC12266406; doi:10.1371/journal.pone.0327989)
Supplement: S1 File — (PDF) [file pone.0327989.s001.pdf]

Questionnaire

Please read each of the following statements carefully and indicate to what extent it corresponds with your behaviors, thoughts, and feelings. How well does each statement describe you? Please be honest. There are no right or wrong answers. The survey is completely anonymous and serves scientific purposes.

	Strongly disagree	Disagree	Somewhat disagree	Somewhat agree	Agree	Strongly agree
(1) I often find myself doing things that contradict my personal values.	○	○	○	○	○	○
(2) When I am not sure that I will succeed (e.g. on an exam), instead of working for it, I take actions (e.g. cleaning, playing) that will justify a possible failure.	○	○	○	○	○	○
(3) When I treat someone badly, I think they deserve it because they are worse than me.	○	○	○	○	○	○
(4) I explain extremely different phenomena by the same cause.	○	○	○	○	○	○
(5) My decisions are often random and then I bend reality to justify my choice.	○	○	○	○	○	○
(6) I attribute my own unapproved characteristics to people whom I like and who are similar to me.	○	○	○	○	○	○
(7) Anonymity makes me more likely to violate moral or social norms.	○	○	○	○	○	○
(8) When I behave aggressively towards a person, it increases the risk of being aggressive again towards that person and towards other people.	○	○	○	○	○	○
(9) I tend to see myself as playing a more central role in events than it actually is.	○	○	○	○	○	○
(10) When I work with others in a group, I overestimate my contribution to the work.	○	○	○	○	○	○
(11) When a phenomenon concerns me, I overestimate its universality, and when it does not concern me, I underestimate its universality.	○	○	○	○	○	○
(12) I often do something just because other people do it.	○	○	○	○	○	○

Kwestionariusz

Proszę przeczytaj uważnie każde z poniższych stwierdzeń i określ, w jakim stopniu jest ono zgodne z Twoimi zachowaniami, myślami, uczuciami. Na ile każde stwierdzenie opisuje Ciebie. Prosimy o szczerość. Nie ma tu odpowiedzi dobrych ani złych. Badanie jest w pełni anonimowe i służy celom naukowym.

	Zdecydowanie nie zgadzam się	Nie zgadzam się	Raczej nie zgadzam się	Raczej zgadzam się	Zgadzam się	Zdecydowanie zgadzam się
(1) Przypisuję własne nieaprobowane cechy lubianym i podobnym do mnie osobom.	○	○	○	○	○	○
(2) Gdy nie mam pewności, że odniosę sukces (np. na egzaminie), zamiast na niego zapracować, podejmuję działania (np. sprzątam, bawię się), które usprawiedliwią ewentualną porażkę.	○	○	○	○	○	○
(3) Gdy kogoś źle traktuję uważam, że on na to zasługuje, bo jest gorszy ode mnie.	○	○	○	○	○	○
(4) Tłumaczę skrajnie różne zjawiska tą samą przyczyną.	○	○	○	○	○	○
(5) Moje decyzje często są przypadkowe, a później naginam rzeczywistość tak, by uzasadnić swój wybór.	○	○	○	○	○	○
(6) Raczej rzadko zachowuję się zgodnie ze swoimi poglądami i przekonaniami.	○	○	○	○	○	○
(7) Anonimowość powoduje, że jestem bardziej skłonny (skłonna) zachować się niezgodnie z normami moralnymi czy społecznymi.	○	○	○	○	○	○
(8) Gdy zachowam się agresywnie wobec jakiejś osoby, zwiększa to ryzyko mojej ponownej agresji wobec tej samej osoby oraz wobec innych osób.	○	○	○	○	○	○
(9) Mam tendencję do spostrzegania siebie jako osoby odgrywającej bardziej centralną rolę w wydarzeniach, niż to jest w istocie.	○	○	○	○	○	○
(10) Gdy pracuję z innymi w grupie przeceniam swój wkład w pracę.	○	○	○	○	○	○
(11) Kiedy jakieś zjawisko mnie dotyczy, przeceniam jego powszechność, a gdy mnie nie dotyczy, nie doceniam jego powszechności.	○	○	○	○	○	○
(12) Często robię coś tylko dlatego, że inni to robią.	○	○	○	○	○	○
