# Supplementary material for: Development and validation of the Self-Awareness of Ego-Threatening Biases Questionnaire (SAETBQ)
Source: PLoS One. 2025 Jul 16;20(7):e0327989. doi: 10.1371/journal.pone.0327989 (PMC12266406; doi:10.1371/journal.pone.0327989)
Supplement: S2 File — OSF Link: https://osf.io/vhwb5/?view_only=185af0d49b2946c2b370b2289221494e. (PDF) [file pone.0327989.s002.pdf]

Supplementary Materials

Development and Validation of the Self-Awareness of Ego-Threatening Biases Questionnaire (SAETBQ)

https://osf.io/vhwb5/?view_only=185af0d49b2946c2b370b2289221494e

OSF repository files overview

This repository contains supplementary materials, data files, and analysis scripts for our questionnaire validation study.

Repository structure

```
/
|-- SAETBQ (ENG and PL).pdf       # Final 12-item version of the SAETBQ
|-- supplementary_materials.pdf   # Main supplementary document
|-- Study1/                       # Study 1 files
    |-- study1.csv                # Raw data
    |-- training_indices.csv      # Train/test split indices
    |-- Study1.R                  # Main analysis script
|-- Study2/                       # Study 2 files
    |-- study2.csv                # Raw data
    |-- Study2.R                  # Analysis script
|-- Additional files/
    |-- bootstrap.R               # Bootstrap script
    |-- bootstrap_factor1.rds     # Pre-fitted bootstrap 1-factor
    |-- bootstrap_factor2.rds     # Pre-fitted bootstrap 2-factor
    |-- bootstrap_factor3.rds     # Pre-fitted bootstrap 3-factor
    |-- bootstrap_factor4.rds     # Pre-fitted bootstrap 4-factor
    |-- factor_comparison.R       # Multiple factor CFAs
```

File descriptions

Study 1 files: The study1.csv file contains raw survey data for 55 preliminary items. The training_indices.csv file provides the 80%-20% training-test split indices used in our cross-validation. Study1.R contains the complete questionnaire validation pipeline including data screening, item selection, and confirmatory factor analysis.

Study 2 files: The study2.csv file contains data for the second validation study. Study2.R includes analyses from the second study.

Both Study1.R and Study2.R files include a user configuration section at the beginning that allows the specification of data paths and analysis parameters, as well as a result display section at the end. To replicate our results, run the Study1.R/Study2.R script using the provided datasets and training indices.

Additional files: We also include bootstrap.R, which runs bootstrap analyzes for competing factor models (2, 3, and 4 factors). Pre-fitted bootstrap results are available in bootstrap_factor1.rds through bootstrap_factor4.rds files. The factor_comparison.R script runs and displays CFA results for multiple factor models. These additional analyses are not part of the main studies, but are included for completeness.

Software information

All statistical analyzes conducted in this study were performed using the R programming language version 4.4.1 R Core Team (2024) on a Lenovo Yoga Slim 7-14 X Elite (model: X1E-78-100/32GB/1TB) running Windows 11 x64 (build 26100).

The following R packages were used for data analysis:

- **readr** (version 2.1.5) Wickham et al. (2024b)
- **dplyr** (version 1.1.4) Wickham et al. (2024a)
- **psych** (version 2.3.9) Revelle (2024)
- **corrplot** (version 0.95) Wei & Simko (2024)
- **GPArotation** (version 2024.3-1) Bernaards & Jennrich (2024)
- **lavaan** (version 0.6-19) Rosseel (2012)
- **semPlot** (version 1.1.6) Epskamp (2022)
- **semTools** (version 0.5-6) Jorgensen et al. (2022)

Study 1: Questionnaire validation pipeline

In our study, we developed a questionnaire validation pipeline, aimed at refining a preliminary set of 55 items (see *Selection of biases* section of the main manuscript). Our pipeline involves: a data screening procedure to ensure data quality; training-test data split; exploratory factor analysis (EFA) item selection on the training dataset; a cross-validation using confirmatory factor analysis (CFA) on the test dataset; and the validation of selected items against related psychological constructs using the full dataset.

Data screening

Our data screening approach identifies and removes responses that exhibit suspicious response patterns. These patterns often manifest as repetitive or invariant responses in the questionnaire, suggesting that some participants might not have mindfully engaged with the survey but rather responded automatically.

We created a custom function (see the **flag** function in the Study1.R file) that analyzes the response patterns within each participant's data and flags those that show unusually low variance (below a threshold of 0.1) or a high degree of uniformity (the proportion of the most common response exceeded 95%). This approach was balanced to flag only those responses strongly indicative of non-discriminatory answering behavior. Consequently, using these stringent thresholds, the function identified and removed only four responses from our dataset.

Cross-validation split

We allocated 80% of our sample to the training dataset. Exploratory analyzes and item selection were performed exclusively on the training subset. The remaining 20% constituted the test dataset, which was reserved for confirmatory analyzes (Osborne & Fitzpatrick, 2012). The final validation of our questionnaire with other psychological constructs was carried out on the combined training and test datasets (full set).

The indices in the provided replication code need to be loaded in the user configuration section of the code at the beginning using the appropriate function for loading the training indices (see configurations in the Study1.R file). The specific train and test splits used in this study are available as **training_indices.csv**. Although our approach is robust enough and should result in the same items being extracted consistently, using a different split might lead to slightly different results (e.g. minor variations in loadings or model fit).

Item selection

We first performed an exploratory factor analysis (EFA) on the full training dataset to assess the loadings of all 55 items onto a single factor. We used a bootstrapped approach with 2000 iterations to ensure the stability of our initial factor loading estimates (Royston & Sauerbrei, 2009). Table 1 presents these initial loadings, which provided a preliminary indication of item relevance.

Table 1: Factor loadings from initial bootstrapped EFA on training dataset

**Item**	**Loading**	**Item**	**Loading**	**Item**	Loading
BN1	0.264	BN20	0.035	BN39	0.384
BN2	0.374	BN21	0.073	BN40	0.444
BN3	0.139	BN22	0.396	BN41	0.291
BN4	0.182	BN23	0.301	**BN42**	**0.497**
BN5	0.257	BN24	-0.045	BN43	0.438
BN6	0.256	BN25	0.385	BN44	0.451
BN7	0.408	**BN26**	**0.556**	BN45	0.322
BN8	0.369	BN27	0.444	BN46	0.267
BN9	0.208	BN28	0.433	**BN47**	**0.500**
BN10	0.371	BN29	0.454	BN48	0.438
BN11	0.271	**BN30**	**0.469**	**BN49**	**0.482**
BN12	0.397	**BN31**	**0.572**	BN50	0.430
BN13	0.260	**BN32**	**0.578**	BN51	0.455
BN14	0.365	**BN33**	**0.605**	BN52	0.043
BN15	0.318	BN34	0.450	BN53	0.421
BN16	-0.011	BN35	0.215	BN54	0.368
BN17	0.122	**BN36**	**0.488**	**BN55**	**0.536**
BN18	0.342	**BN37**	**0.470**		
BN19	0.338	**BN38**	**0.503**		

For the selection of final items, we followed the recommendations of Capanu et al. (2020) and De Bin et al. (2015), who advocate repeated data splitting as a superior approach to assess the stability of the parameters. This process involved conducting EFA on multiple internal splits of the training dataset, repeated across 1000 iterations. In each iteration, 60% of the training dataset, randomly selected, was used to assess the loadings of the 55 items on a single factor. Items that met or exceeded a loading threshold of 0.45 were retained.

We created a custom function (see the **cleaning_efa** function in the Study1.R file) that performs EFA on a dataset and filters out items that do not meet the specified loading threshold. This function was then applied across multiple data splits to identify the most stable items. The frequency of selection for each item was calculated in these iterations, with items retained in at least 80% of the iterations deemed the most robust. This repeated data splitting procedure ensures that the final items are stable, reflecting true characteristics of the construct being measured, rather than anomalies in the data splits. Without this, there is a risk of including items that appear relevant due to random variations of a single dataset, rather than their true relevance to the construct.

The repeated data splitting analysis revealed varying levels of robustness across items. Table 2 presents the proportion of iterations in which each item exceeded the loading threshold of 0.45. Of the initial set of 55 items, only 12 were retained in more than 80% of the iterations: **BN26, BN30, BN31, BN32, BN33, BN36, BN37, BN38, BN42, BN47, BN49, BN55**. These 12 selected items correspond to the top 12 items with the highest loadings from our initial bootstrapped analysis (see bolded items in Table 1), further validating the robustness of our selection approach.

Table 2: Proportion of iterations where items exceeded loading threshold 0.45

Item	Proportion	Item	Proportion	Item	Proportion
BN26	1.000	BN49	0.943	BN48	0.288
BN31	1.000	BN37	0.851	BN43	0.277
BN32	1.000	BN30	0.810	BN28	0.213
BN33	1.000	BN51	0.597	BN50	0.170
BN55	1.000	BN29	0.572	BN53	0.091
BN38	0.995	BN44	0.512	BN7	0.038
BN47	0.992	BN34	0.511	BN12	0.014
BN42	0.988	BN40	0.406	BN22	0.008
BN36	0.966	BN27	0.395	Other items	< 0.004

Note. The "Other items" category includes BN25, BN39, and BN54, each with proportions less than or equal to 0.004.

Confirmatory Factor Analysis

Following the item selection process, we conducted a Confirmatory Factor Analysis (CFA) on the test dataset to validate the items identified in the item selection step. The final set of 12 items, derived from the iterative EFA, was specified as indicators of a one-factor structure. We chose the Robust Maximum Likelihood (MLR) estimator for our CFA. MLR is appropriate for handling the non-normal distribution of Likert scale data, providing more accurate estimates than other estimators that assume continuous and normally distributed variables.

The CFA results demonstrate an exceptionally good model fit. The fit indices, comprising the Comparative Fit Index (CFI), Tucker-Lewis Index (TLI), Root Mean Square Error of Approximation (RMSEA), and Standardized Root Mean Square Residual (SRMR), exceeded the commonly accepted thresholds for a good fit ($\chi^2 = 112.26$, $df = 54$, $p < .001$, $CFI(robust) = .950$, $TLI(robust) = .939$, $RMSEA(robust) = .045$, $SRMR(robust) = .045$). The CFI and TLI values were notably high, surpassing the 0.90 threshold, while the RMSEA and SRMR values fell well below the 0.05 upper limit, indicative of an excellent fit.

Comparing one factor solution to multiple factor solutions

The one factor solution is the theoretically appropriate solution as indicated in the main text of the manuscript. However, we also explored other potential multi-factor models. Our justification for this additional analysis comes from the results of initial data exploration using Very Simple Structure analysis and EFA on the full dataset. This initial analysis yielded the results presented in Table 3.

Table 3: Selected fit statistics by number of factors

Factors	vss1	vss2	χ^2/df	RMSEA	BIC	SRMR
1	0.62	0.00	5.23	0.051	-3085	0.063
2	0.54	0.70	3.49	0.039	-5352	0.042
3	0.44	0.67	2.68	0.032	-6221	0.033
4	0.36	0.62	2.12	0.026	-6684	0.026

Although Table 3 indicates that the four-factor solution produces the best fit indices, these statistics were calculated with all 55 items (including those with weak or diffuse loadings) so the apparent superiority of this model may reflect overfitting rather than a genuinely stronger latent structure (Goretzko & Bühner, 2021). However, this result motivated a more rigorous evaluation with our pipeline approach.

We conducted the initial bootstrap exploratory factor analysis for two, three, and four factor models on the train dataset. Each bootstrap comprised 2000 iterations. We used promax rotation to allow our factors to correlate. From the estimated bootstrap factor loadings, we selected those that exceeded 0.45. This procedure resulted in the items loading on the factors presented in Table 4.

Table 4: Items loading on factors across different factor models

Factor	1-Factor	2-Factor*	3-Factor	4-Factor
F1	BN26, BN30, BN31, BN32, BN33, BN36, BN37, BN38, BN42, BN47, BN49, BN55	BN12, BN26, BN31, BN32, BN33, BN34, BN36, BN37, BN38, BN40, BN42, BN47, BN50, BN55	BN31, BN32, BN33, BN34, BN36, BN40, BN47, BN50, BN55	BN31, BN35, BN37, BN38, BN42, BN43, BN45, BN46, BN47, BN48, BN49
F2	—	BN9, BN21	BN6, BN8, BN9, BN18	BN6, BN8, BN9, BN18
F3	—	—	BN46	BN21, BN24
F4	—	—	—	BN12, BN14

* Item BN24 had loading exceeding 0.45 on the second factor but was excluded because the CFA model failed to converge when this item was included.

This item composition reveals that multiple factor solutions are inadequate. The multiple factor structures that emerged consistently featured one large factor comprising most items (ranging from 14 items in the 2-factor model to 9 items in the 3-factor model). The smaller factors consisted of 4 to as few as 1 item. There is no substantive justification for maintaining a separate factor with only one or two items. Attempting to attribute meaningful interpretation to such factors represents a futile exercise. The bootstrapped EFA for multiple factor solutions is available in the bootstrap.R file.

Table 5: Comparison of fit indices across different factor models (test data)

Fit Index	1-Factor	2-Factor	3-Factor	4-Factor
χ^2 (df)	112.26 (54)	211.34 (103)	168.34 (75)	323.58 (146)
χ^2/df	2.08	2.05	2.24	2.22
CFI	0.935	0.897	0.892	0.829
TLI	0.920	0.880	0.869	0.800
Robust CFI / TLI	0.950 / 0.939	0.913 / 0.899	0.901 / 0.880	0.844 / 0.818
RMSEA (90% CI)	0.058 (0.043–0.073)	0.057 (0.046–0.068)	0.062 (0.050–0.075)	0.061 (0.052–0.070)
Robust RMSEA	0.050 (0.031–0.069)	0.052 (0.039–0.065)	0.059 (0.045–0.073)	0.058 (0.048–0.068)
SRMR	0.045	0.055	0.061	0.061
AIC	13,078.98	17,621.97	15,433.91	21,104.65
BIC	13,169.57	17,746.53	15,547.15	21,270.73

Nevertheless, we conducted confirmatory factor analysis for all multiple factor models on the test dataset and compared them to the one factor structure. The model comparison results appear in Table 5. The results demonstrate that the one-factor model exhibits a better fit across most indices. The one-factor model

displays the highest CFI (0.935) and TLI (0.920) values, with robust versions reaching 0.950 and 0.939 respectively. Although the RMSEA values are comparable across models, the one-factor model presents the lowest robust RMSEA (0.05) and SRMR (0.045). Furthermore, the one-factor model shows substantially lower AIC and BIC values, suggesting that it provides the most parsimonious representation of the data structure. Moreover, the two-factor structure exhibited convergence issues, with item BN24 loading above 0.45 on the second factor but causing the model to not converge when included. Even with this item excluded, we observed problematic negative variances (e.g., BN9: -1799.550), further suggesting fundamental issues with the two-factor solution. When considered alongside the theoretical rationale presented in the main manuscript and the problematic structure of multi-factor solutions (with several factors containing only 1-2 items), these results offer compelling support for adopting the one-factor model as the optimal representation of our construct.

References

Bernaards, C. A., & Jennrich, R. I. (2024). GPArotation: Gradient projection algorithms for factor rotation (Version 2024.3-1) [Computer software]. Retrieved from https://CRAN.R-project.org/package=GPArotation

Bernaards, C. A., & Jennrich, R. I. (2005). Gradient projection algorithms and software for arbitrary rotation criteria in factor analysis. *Educational and Psychological Measurement, 65*(5), 676–696. https://doi.org/10.1177/0013164404272507

Capanu, M., Giurcanu, M., Begg, C. B., & Gönen, M. (2020). Optimized variable selection via repeated data splitting. *Statistics in Medicine, 39*(16), 2167–2184. https://doi.org/10.1002/sim.8538

De Bin, R., Janitza, S., Sauerbrei, W., & Boulesteix, A. (2015). Subsampling versus bootstrapping in Resampling-Based model selection for multivariable regression. *Biometrics, 72*(1), 272–280. https://doi.org/10.1111/biom.12381

Epskamp, S. (2022). semPlot: Path diagrams and visual analysis of various SEM packages' output (Version 1.1.6) [Computer software]. Retrieved from https://CRAN.R-project.org/package=semPlot

Goretzko, D., & Bühner, M. (2021). Robustness of factor solutions in exploratory factor analysis. *Behaviormetrika, 49*(1), 131–148. https://doi.org/10.1007/s41237-021-00152-w

Jorgensen, T. D., Pornprasertmanit, S., Schoemann, A. M., & Rosseel, Y. (2022). semTools: Useful tools for structural equation modeling (Version 0.5-6) [Computer software]. Retrieved from https://CRAN.R-project.org/package=semTools

Osborne, J. W., & Fitzpatrick, D. C. (2012). Replication analysis in exploratory factor analysis: What it is and why it makes your analysis better. *Practical Assessment, Research & Evaluation, 17*(15), 1–8. https://doi.org/10.7275/h0bd-4d11

R Core Team. (2024). R: A language and environment for statistical computing (Version 4.4.1) [Computer software]. R Foundation for Statistical Computing, Vienna, Austria. Retrieved from https://www.R-project.org/

Revelle, W. (2024). psych: Procedures for psychological, psychometric, and personality research (Version 2.3.9) [Computer software]. Northwestern University. Retrieved from https://CRAN.R-project.org/package=psych

Rosseel, Y. (2012). lavaan: An R package for structural equation modeling. *Journal of Statistical Software, 48*(2), 1-36. https://doi.org/10.18637/jss.v048.i02

Royston, P., & Sauerbrei, W. (2009). Bootstrap assessment of the stability of multivariable models. *The Stata Journal Promoting Communications on Statistics and Stata, 9*(4), 547–570. https://doi.org/10.1177/1536867x0900900403

Wei, T., & Simko, V. (2024). corrplot: Visualization of a correlation matrix (Version 0.95) [Computer software]. Retrieved from https://github.com/taiyun/corrplot

Wickham, H., François, R., Henry, L., Müller, K., & Vaughan, D. (2024a). dplyr: A grammar of data manipulation (Version 1.1.4) [Computer software]. Retrieved from https://CRAN.R-project.org/package=dplyr

Wickham, H., Hester, J., & Bryan, J. (2024b). readr: Read rectangular text data (Version 2.1.5) [Computer software]. Retrieved from https://CRAN.R-project.org/package=readr
